# Parasitization by *Cotesia chilonis* Influences Gene Expression in Fatbody and Hemocytes of *Chilo suppressalis*

**DOI:** 10.1371/journal.pone.0074309

**Published:** 2013-09-23

**Authors:** Shun-Fan Wu, Fang-Da Sun, Yi-Xiang Qi, Yao Yao, Qi Fang, Jia Huang, David Stanley, Gong-Yin Ye

**Affiliations:** 1 State Key Laboratory of Rice Biology and Key Laboratory of Agricultural Entomology of Ministry of Agriculture, Institute of Insect Sciences, Zhejiang University, Hangzhou, Zhejiang, China; 2 United States Department of Agriculture/Agricultural Research Service, Biological Control of Insects Research Laboratory, Columbia, Missouri, United States of America; Onderstepoort Veterinary Institute, South Africa

## Abstract

**Background:**

During oviposition many parasitoid wasps inject various factors, such as polydnaviruses (PDVs), along with eggs that manipulate the physiology and development of their hosts. These manipulations are thought to benefit the parasites. However, the detailed mechanisms of insect host-parasitoid interactions are not fully understood at the molecular level. Based on recent findings that some parasitoids influence gene expression in their hosts, we posed the hypothesis that parasitization by a braconid wasp, *Cotesia chilonis*, influences the expression of genes responsible for development, metabolism and immune functions in the fatbody and hemocytes of its host, *Chilo suppressalis*.

**Methodology/Principal Findings:**

We obtained 39,344,452 reads, which were assembled into 146,770 scaffolds, and 76,016 unigenes. Parasitization impacted gene expression in fatbody and hemocytes. Of these, 8096 fatbody or 5743 hemocyte unigenes were down-regulated, and 2572 fatbody or 1452 hemocyte unigenes were up-regulated. Gene ontology data showed that the majority of the differentially expressed genes are involved in enzyme-regulated activity, binding, transcription regulator activity and catalytic activity. qPCR results show that most anti-microbial peptide transcription levels were up-regulated after parasitization. Expression of bracovirus genes was detected in parasitized larvae with 19 unique sequences identified from six PDV gene families including ankyrin, CrV1 protein, cystatin, early-expressed (EP) proteins, lectin, and protein tyrosine phosphatase.

**Conclusions:**

The current study supports our hypothesis that parasitization influences the expression of fatbody and hemocyte genes in the host, *C. suppressalis*. The general view is that manipulation of host metabolism and immunity benefits the development and emergence of the parasitoid offsprings. The accepted beneficial mechanisms include the direct impact of parasitoid-associated virulence factors such as venom and polydnavirus on host tissues (such as cell damage) and, more deeply, the ability of these factors to influence gene expression. We infer that insect parasitoids generally manipulate their environments, the internal milieu of their hosts.

## Introduction

Parasitoid wasps of the order Hymenoptera develop as parasites of other arthropods during their larval stages, giving rise to free-living adults. They are valued biological control agents for various insect pests [1]. Endoparasitoid wasps (whose larvae develop inside, rather than, on their hosts) introduce substances into their hosts during oviposition, including venom, polydnaviruses (PDVs), ovary fluids, and other maternal factors; these materials act to ensure successful development of their progeny [2]. These factors influence host behavior [3], metabolism, development [4], endocrine system activity [5] and immune defense reactions [1], [6], [7]. There are over 100,000 host-parasitoid systems and most of them are shaped by differing selective forces [6]. These co-evolved systems have produced an unknown, but large number of variations on the broad theme of molecular host-parasitoid interactions. Only a few of these relationships have been deeply investigated, and much more knowledge is required to generate broad principles of molecular parasitoid-host systems.

The rice stem borer, *Chilo suppressalis* (Walker) (Lepidoptera: Crambidae) is a destructive rice pest in China and other Asian countries. It is responsible for severe crop loss every year, especially in China because of changes in rice cultivation and the popularization of hybrid rice. Hybrid strains are more susceptible to insect damage than other rice releases [8]. *C. suppressalis* has developed resistance to many groups of chemical insecticides [9], [10] and the estimated cost of controlling this pest is around 1 billion yuan annually [11]. Crop damage and high resistance emphasizes the urgency for developing innovative control measures and resistance management strategies. Parasitoids or parasitoid-produced regulatory molecules have the potential to improve conventional pest control strategies in ways that supports sustainable agriculture [12]. *Cotesia chilonis* (Matsumura) (Hymenoptera: Braconidae), mainly distributed in southeastern and eastern parts of Asia, is the major endoparasitoid of *C. suppressalis* larvae [13]. *C. chilonis* injects venom, PDV and teratocytes as major parasitoid-associated factors while ovipositing into hosts [14]. The injected virus is in the genus *Bracovirus* (BV) (Family: Polydnaviridae) similar to *Cotesia vestalis* [15]. The biological characteristics of *C. chilonis* and its effects on the immune response of *C. suppressalis* larvae has been preliminarily investigated [16]. When dissecting the parasitized hosts, we found that the egg matured in 2 d, and larvae seemed to have three instars, the first two instar ones molted inside the host, and the third instar ones emerged from the host to spin a cocoon. The first, second, and third instar lasted 2, 3, and 1 d at 25±1°C and 60∼65% relative humidity, respectively. The pupae develop for 3 d. After parasitization by *C. chilonis*, total amount of food consumption of host larvae, compared with non-parasitized larvae, reduced by 36.75%. During the parasitization, the host development rate was restrained and the times of host-mounting become less, and the host larvae could not develop into pupae stage [17]. Parasitization by *C. chilonis* may also result in some regular changes of immunity of its host *C. suppressalis* [18]. For example, total number of hemocytes in parasitized larvae became significantly higher than that of non-parasitized (n.p.) control from 1 day post-parasitization (p.p.) [18].

Parasitoid wasps have evolved an array of mechanisms to regulate the host’s physiology and biochemistry in a way that creates a microenvironment for successful development [6]. Previous studies have concentrated mainly on individual or small defined groups of host genes to explore their functions or differential expression following parasitization [19]–[21]. Only a few studies report on large-scale approaches to understanding the global impacts of parasitization on hosts at the genome level. Using suppression subtractive hybridization, Fang et al. [22] found that *Pteromalus puparum* venom treatments led to reductions in expression of a large number of immune-related genes in the lepidopteran host *Pieris rapae.* Gene expression changes in flour moth *Ephestia kuehniella* caterpillars after parasitization by the endoparasitic wasp *Venturia canescens* were analyzed using cDNA-amplified fragment length polymorphisms, which demonstrated that expression of 13 transcripts in parasitized hosts were suppressed by the wasp [23]. Deep sequencing-based transcriptome analysis of *Plutella xylostella* larvae parasitized by *Diadegma semiclausum* also indicated that parasitization had significant impacts on expression levels of 928 identified insect host transcripts [12].

In the present study, we used the Illumina sequencing technology to explore the *C. suppressalis* gene expression changes induced by *C. chilonis* parasitization. We first obtained and characterized the transcriptome of *C. suppressalis* larvae parasitized by *C. chilonis*. A systematic bioinformatics strategy was engaged to functional annotation of the transcriptome. Additionally, we constructed four RNA-seq (quantification) and compared the accumulation of transcription products of fat body and hemocytes in non-parasitization (n.p.) versus post-parasitization (p.p.) hosts, *C. suppressalis*. The results give us a comprehensive view of global gene expression profiles of two immune-related tissues of host response to parasitization, and establish a sound foundation for future molecular studies based on high throughput sequence data.

## Materials and Methods

### Insect rearing, parasitization and RNA isolation

*C. suppressalis* were reared on artificial diet [24]. The wasps, *C. chilonis,* were reared on host larvae. Both species were maintained at 25±1°C under natural photoperiod and relative humidity approximately 80%. To obtain material for sequencing, 100 larvae with the age of day 2 (4^th^ instar) were exposed to a mated female wasp until parasitization was observed. Individual parasitized larvae were maintained on artificial diet under the conditions described until tissue samples were prepared.

Larvae of *C. suppressalis* were surface-sterilized with 70% ethanol. Hemocytes were prepared by puncturing a proleg and allowing hemolymph to freely drip into insect Grace’s medium (1:10, v/v; Invitrogen, Carlsbad, CA) in 1.5 ml chilled Eppendorf tubes and centrifuged at 200 × g for 10 min at 4°C After centrifugation, plasma was discarded and hemocytes were used for total RNA extraction. The fat bodies were removed from the remaining cadaver under a stereomicroscope and transferred into phosphate-buffered saline (NaCl 137 mM, KCl 2.7 mM, Na_2_HPO_4_ 10 mM, KH_2_PO_4_ 2 mM, pH 7.2 ∼ 7.4) in 1.5 ml Eppendorf tubes. Total RNA samples were extracted using TRIZOL Reagent (Invitrogen) following the manufacturer’s instructions and stored in –80°C. RNA sample concentrations were determined using an Agilent 2100 Bioanalyzer (Agilent Technologies, Palo Alto, CA). Integrity was ensured through analysis on a 1.5% (w/v) agarose gel.

### Transcriptome analysis library preparation and sequencing

The previous of our work showed that the immune indices like hemocyte spreading rate, mortality, phagocytic rate, encapsulation index and phenoloxidase activity were all significantly changed after parasitism in 0.5 to 2 days [18]. Besides this, immature development of *C. chilonis* was studied by dissecting parasitized hosts in the laboratory at 25±1°C and 60 – 65% RH. When dissecting the parasitized hosts, we found that the egg matured in 2 d [14]. Hence, we selected 6, 12, 24 and 48 hrs p.p. based on the influences of *C. chilonis* development on host immunity [18]. The purpose of these four time intervals was to obtain a comprehensive sampling of transcripts, some of which would have been missed if tissues were collected at a single time point. Cs-FB and Cs-HC RNA was prepared at the same time as fat body and hemocytes from parasitized larvae (PCs-FB; PCs-HC) from day 2, 4th instar naïve larvae (100). To obtain complete gene expression information, a pooled RNA sample including sixteen RNA samples composed of four time points (6, 12, 24 and 48 h) of four treatments (PCs-FB, PCs-HC, Cs-FB and Cs-HC) was used for transcriptome analysis.

The cDNA library was prepared according to the Illumina manufacturer’s instructions. Briefly, oligo (dT) beads were used to isolate poly(A) mRNA from total RNA (pooled RNA of control and experimental fat body and hemocytes). Short mRNA fragments were created by adding fragmentation buffer. Then, first and second strand cDNA were synthesized from cleaved RNA fragments. Short fragments were purified with QiaQuick PCR extraction kits (Qiagen, Hilden, Germany) and resolved with EB buffer for end reparation and adding poly(A). The short fragments were connected to sequencing adapters. Following agarose gel electrophoresis, suitable fragments were selected for PCR amplification as templates. The library was sequenced using Illumina HiSeq™ 2000 (Illumina, Inc, San Diego, CA) at Beijing Genomics Institute (BGI)-Shenzhen, China (http://www.genomics.cn).

### *De novo* transcriptome assembly and unigene annotation

The raw reads from the images and quality value calculation were performed by the Illumina data processing pipeline (version 1.6). After removal of low quality reads, clean reads were assembled into sequence contigs, scaffolds, and unigenes using the short reads assembling program SOAPdenovo [25]. All raw sequencing data have been deposited in NCBI’s Short Read Archive (SRA) database (http://www.ncbi.nlm.nih.gov/sra) under accession number: SRR651040. The Transcriptome Shotgun Assembly (TSA) project has been deposited at DDBJ/EMBL/GenBank under the accession number: GAJS00000000.

The unigenes were used for BLAST search and annotation against NR database and Swissprot database with an E-value cut of E-value^−5^. Gene ontology (GO) and Kyoto Encyclopedia of Genes and Genomes (KEGG) Orthology (KO) annotations of the unigenes were determined using Blast2GO and Blastall software [26].

### RNA-seq (quantification) library preparation, sequencing and alignment with references

Total RNAs of four time points were mixed equally to create one library. Therefore, four RNA-seq libraries including, PCs-FB, PCs-HC, Cs-FB and Cs-HC, were prepared. The RNA-seq sequencing method was the same with transcriptome analysis (transcriptome analysis library preparation and sequencing). Briefly, after filter procedures, we obtained the clean reads, which were the basis of all following analysis. For the BGI bioinformatics pipeline, clean reads from each library were separately mapped against the reference set of assembled transcripts using SOAPaligner/soap2 [25]. Mismatches of no more than 2 bases were allowed in the alignment.

### Gene expression level and differentially expressed genes identification

Gene expression levels were calculated using Reads Per Kilobase per Million (RPKM) mapped reads [27]. If there was more than one transcript for a gene, the longest one was used to calculate its expression level and coverage. Thus, the output for each dataset can be directly compared as the number of mapped reads per dataset and transcript size has been taken into account.

The correlation of the detected count numbers between parallel libraries were assessed statistically by calculating the Pearson correlation. False discovery rate (FDR) was used to determine differentially expressed genes [28]. Assume that we have picked out R differentially expressed genes in which S genes show differential expression and the other V genes are false positives. If the error ratio Q  =  V/R must remain below a cutoff (1%), FDR should not exceed 0.01. In this research, P ≤ 0.01, FDR ≤ 0.001 and the absolute value of log_2_Ratio ≥ 1 were used as threshold values to identify differentially expressed genes [29].

### Quantitative real-time PCR (qRT-PCR) validation

Total RNA was extracted as described for RNA-seq library preparation and sequencing. Following DNAse Ι (RQ1 RNase-free DNase: Promega) treatment, total RNA (1μg) was used for cDNA synthesis with ReverTra Ace qPCR RT kits (Toyobo, Osaka, Japan).. Quantitative RT-PCR (qPCR) reactions (20 µl) were performed in triplicate using SsoFast EvaGreen Supermix with low ROX (BioRad) in a 7500 Real Time PCR System (Applied Biosystems by Life Technologies). The qPCR reaction consisted of 2 µl of diluted cDNA (10 ng) and 1 µM of each primer, which were selected for at least 90% amplification efficiency. The PCR reactions were programmed at 95°C for 30 sec; 40 cycles of 95°C for 5 sec, 60°C for 34 sec, followed by melting curve analysis for quality control (60°C to 95°C). No primer dimer was detected in the melting curves. The data were analyzed using the comparative Ct (ddCt) method [30], and the endogenous 18S rRNA reference gene [31] was used for normalization. At least three replicates were tested per sample.

We performed another experiment to record gene expression levels at 6, 12, 24 and 48 h p.p. for a selected group of genes. For each time point, three independent groups of 30 control larvae and three independent groups of 30 parasitized 4^th^ instar larvae were processed for RNA extraction.

## Results and Discussion

### Illumina sequencing and reads assembly

**Figure 1:**
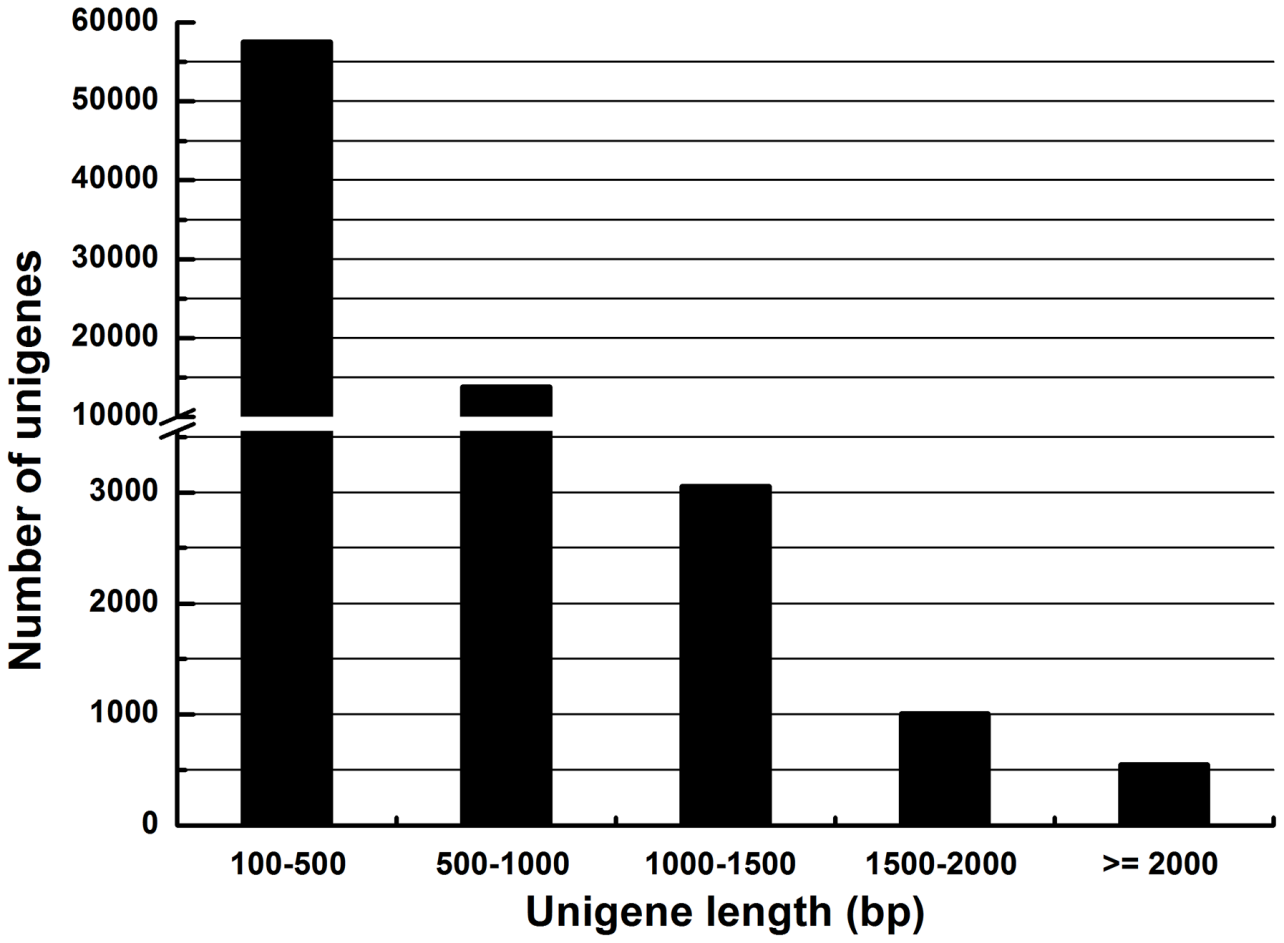
Length distribution of *Chilo suppressalis* unigenes. The histogram bars represent the numbers of unigenes in each length category.

Illumina sequencing resulted in 39,344,452 raw reads, corresponding to an accumulated length of 3,541,000,680 bp (Table 1). The raw reads were assembled into 1,028,924 contigs with a mean length of 127 bp. Using paired end-joining and gap-filling, these contigs were further assembled into 146,770 scaffolds with a mean length of 275 bp. Scaffold sequences were assembled into clusters using TGI software. We obtained 76,016 unigenes with a mean length of 440 bp. The lengths of 18,462 unigenes were ≥ 500 bp and the lengths of the remaining 57,554 unigenes (75% of the total) were between 100 to 500 bp (Figure 1), similar to other insect transcriptome projects using this technology [32], [33].

**Table 1:** Sequence statistics of the Illumina deep sequencing of *Chilo suppressalis* larvae transcriptome.

	Reads	Contigs	Scaffolds	Unigenes
Number of sequences	39,344,452	1,028,924	146,770	76,016
Mean length (bp)	90	127	275	440
Total length (bp)	3,541,000,680	127,183,235	58,271,577	33,412,141

### Annotation of predicted proteins, GO and COG classification

**Figure 2:**
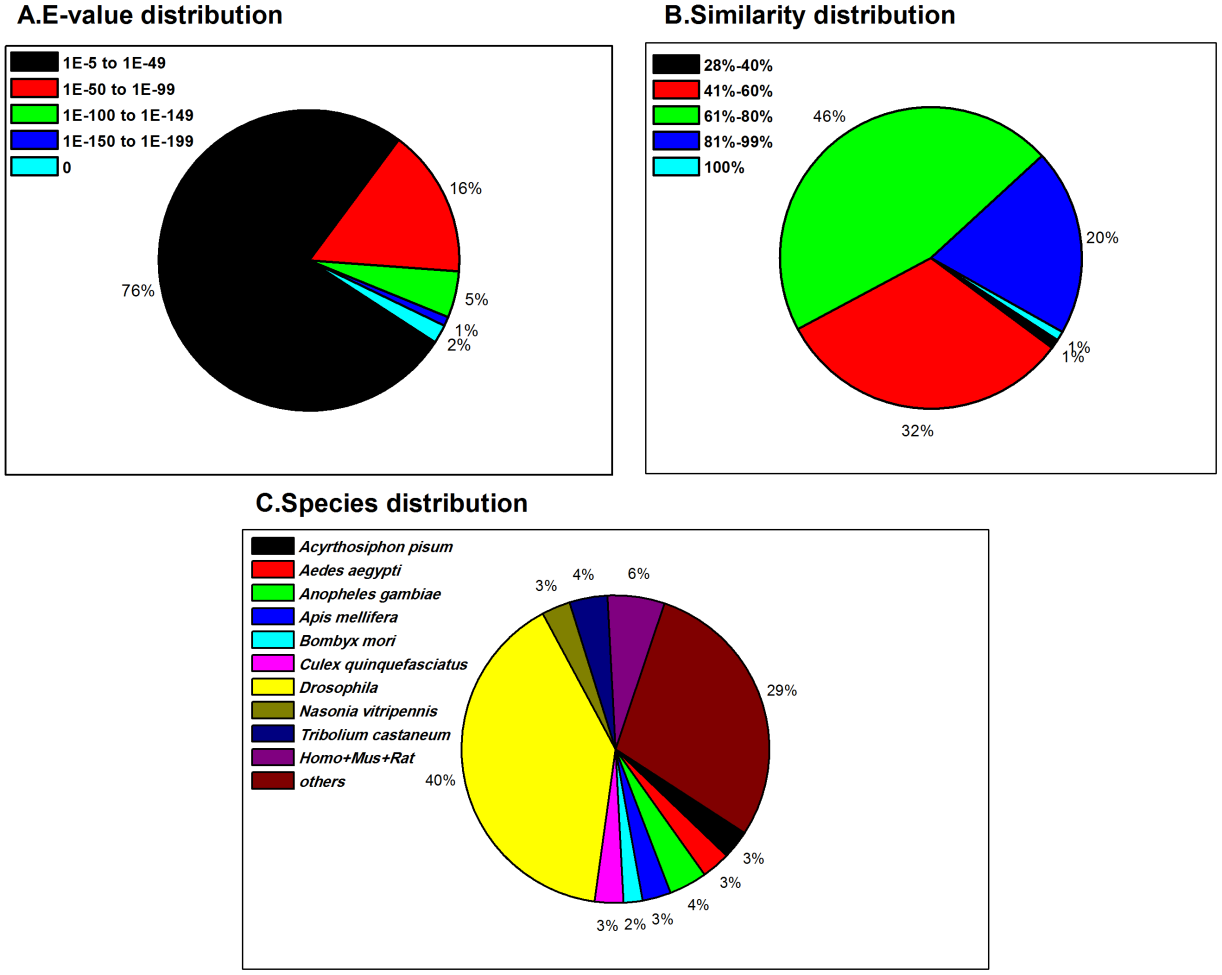
Homology analysis of *Chilo suppressalis* unigenes. (A) E-value distribution of BLAST hits for each unique sequence with cut-off E-value  =  1.0E-5. (B) Similarity distribution of the top BLAST hits for each sequence. (C) Species distribution of the BLASTX results. We used the first hit of each sequence for analysis. Homo: *Homo sapiens*; Mus: *Mus musculus*; Rat: *Rattus norvegicus*. Each slice of the pie-charts represents proportions of the total sequences.

For functional annotation, the 76,016 unigenes were searched using BLASTx, with a threshold of E value < 10^−5^, against four public databases (NCBI non-redundant (nr) database, the Swiss-Prot protein database, the Kyoto Encyclopedia of Genes and Genomes (KEGG) database, and the Clusters of Orthologous Groups (COG) of proteins database. The E-value distribution of the top hits in the nr database showed that 24% of the mapped sequences have strong homology (less than 1.0E^−49^) and 76% of homolog sequences ranged between 1.0E^−5^ to 1.0E^−49^ (Figure 2A). The similarity distribution has a comparable pattern with 21% of the sequences having similarity higher than 80%, while 79% of the hits have similarities ranging from 28% to 80% (Figure 2B). The results are similar to transcriptome analyses of other insect species using this technology [34], [35]. The species distribution of the best match result for each sequence showed that 40% of the *C. suppressalis* sequences match with sequences from the *Drosophila* species, while very low proportion (2%) of them have matches to *Bombyx mori* (Figure 2C). One reason for the higher number of hits against the fruit fly genome is that approximately ten times more *Drosophila* genes than *B. mori* genes are deposited in databases.

In total, 11,886 unigenes were assigned GO terms based on Blast2GO [26] and WEGO [27] software. In each of the 3 main categories of GO classification, biological process (cell process dominates), cellular component (cell part dominates), and molecular function (binding dominates), show the analyzed tissues were most likely undergoing rapid growth and extensive metabolic activities. We did not find genes representing other clusters. We registered a high-percentage of genes from categories of “metabolic process”, “biological regulation” and “catalytic activity” and only a few genes from terms “synapse part” and “antioxidant activity” (Figure S1). We assigned 14,809 unigenes to COG clusters (Figure S2). Among the 25 COG categories, the cluster for “General function prediction” represents the largest group (2587, 17.5%) followed by “Replication, recombination and repair” (1438, 9.7%) and 'Translation, ribosomal structure and biogenesis' (1219, 8.2%). The category of “secondary metabolites biosynthesis, transport and catabolism” (414, 2.8%) was particularly important because of the importance of secondary insecticide metabolites in insects. The most abundant sequences in this category are cytochrome P450 monooxygenases.

### Statistics of RNA-seq (quantification) and differential gene expression

**Figure 3:**
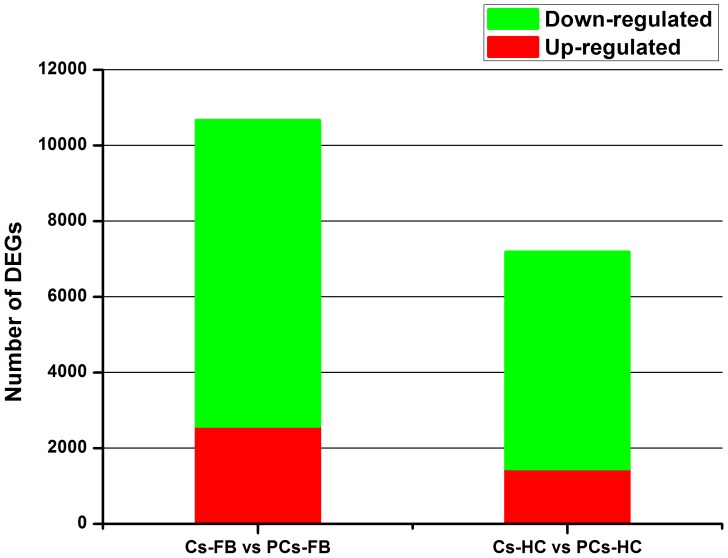
Transcripts differentially expressed between fatbody and hemocytes of non-parasitized and parasitized *Chilo suppressalis* larvae. Up-(red) and down-regulated (green) transcripts were quantified.

To characterize the gene expression profiles of fatbody and hemocytes in parasitized *C. suppressalis* by *C. chilonis*, four RNA-seq (quantification) libraries were constructed and sequenced. We generated 12,052,737 reads from control fat body (Cs-FB), 12,361,322 from parasitized fat body (PCs-FB), 12,466,924 from control hemocytes (Cs-HC) and 11,471,001 from parasitized hemocytes (PCs-HC) (Table 2). These reads were mapped with reference sequences. Our data analyses indicate that parasitism has a significant impact on the gene expression profile of larval fatbody and hemocytes. For fatbody, 10,668 unigenes were differentially expressed after parasitization, with 2,572 (24%) up-regulated and 8,096 (76%) down-regulated. For hemocytes, 7,195 unigenes were differentially expressed after parasitization, with 1,452 (20%) up-regulated and 5,743 (80%) down-regulated (Figure 3). It can be shown that only 14% transcripts of *C. suppressalis* were differentially expressed after parasitization. It indicated that parasitization alter the abundance of a relatively low proportion of *C. suppressalis* transcripts in fat body and hemocytes

**Table 2:** Summary statistics of RNA-seq (quantification) library sequencing and mapping.

Map to gene	Cs-FB	PCs-FB	Cs-HC	PCs-HC
Total reads (percentage)	12052737 (100.00%)	12361322 (100.00%)	12466924 (100.00%)	11471001 (100.00%)
Total base pairs (percentage)	590584113 (100.00%)	605704778 (100.00%)	610879276 (100.00%)	562079049 (100.00%)
Total mapped reads (percentage)	4742642 (39.35%)	4550174 (36.81%)	5785322 (46.41%)	3997504 (34.85%)
Perfect match (percentage)	3614788 (29.99%)	3380066 (27.34%)	4391487 (35.23%)	3028700 (26.40%)
< = 2bp mismatch (percentage)	1127854 (9.36%)	1170108 (9.47%)	1393835 (11.18%)	968804 (8.45%)
Unique match (percentage)	4634561 (38.45%)	4466161 (36.13%)	5630031 (45.16%)	3931673 (34.27%)
Multi-position match (percentage)	108081 (0.90%)	84013 (0.68%)	155291 (1.25%)	65831 (0.57%)
Total unmapped reads (percentage)	7310095 (60.65%)	7811148 (63.19%)	6681602 (53.59%)	7473497 (65.15%)

### GO analysis of differentially expressed unigenes

**Figure 4:**
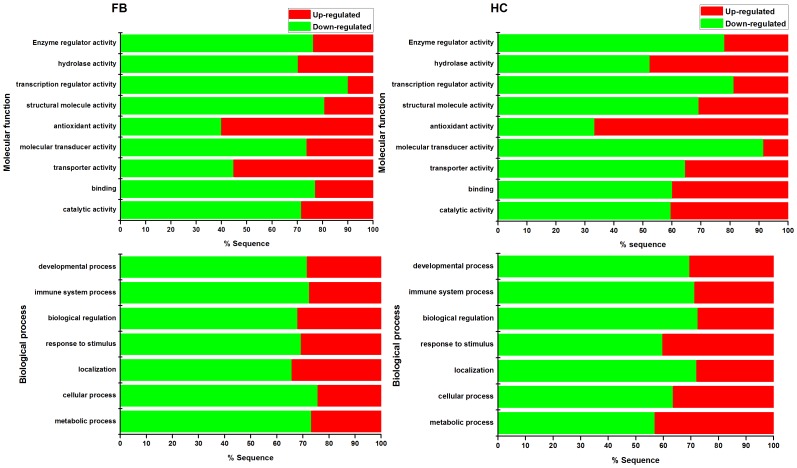
GO term (level 2) enrichment analyses. Selected Go terms from molecular function and biological process, which most related to parasitization, were used in creating diagrams. In molecular function category, one GO terms of antioxidant activity showed the highest up-regulated transcripts both of faybody (FB) and hemocytes (HC). Up-(red) and down-regulated (green) transcripts were quantified.

Most of the differentially expressed transcripts (DETs) for the GO terms, molecular function and biological process, were down-regulated except antioxidant activity (Figure 4). This finding differs from the analysis of *P. xylostella* parasitized by *D. semiclausum* because most of the DETs were up-regulated [12]. One reason may be that this is a species-specific response and another reason may be that different PDV genera are associated with these two parasitoid wasps. *Ichnovirus* (IV) is associated with *D. semiclausum* and PDV with C. *chilonis* belongs to BV. Although viruses in these two genera have similar immunosuppressive and developmental effects on parasitized hosts, they differ morphologically and their encapsidated genomes largely encode different genes [15], [36].

### Transcripts related to immunity

Parasitism exerted significant impact on the transcriptome profile of fatbody and hemocytes. Among the changed unigenes, those related to immunity, development and metabolism are displayed in Table 3 and 4. These transcripts are most relevant to parasitism.

**Table 3:** A list of *Chilo suppressalis* immune-related transcripts that were differentially expressed after parasitization by *Cotesia chilonis*.

Gene family Function	Gene ID	Nt. Length	RPKM				Log2 Ratio		Blast results
			Cs-FB	Cs-HC	PCs-FB	PCs-HC	PCs-FB/Cs-FB	PCs-HC/Cs-HC	
**Pattern recognation receptors**	GAJS01023399	1079	28.8	2.5	12.7	1.9	–1.2	-	gi|113208232|dbj|BAF03520.1|/1.10532e-31/peptidoglycan recognition protein B [*Samia cynthia ricini*]
	GAJS01000005	1943	37	5.9	11.7	0.3	–1.7	–4.5	gi|154689979|ref|NP_001019891.2|/4.04972e-84/hemicentin 1 [*Mus musculus*]
	GAJS01005743	1213	275.9	894.7	1760.2	33265.0	2.7	1.9	gi|52782740|sp|Q8MU95.1|BGBP_PLOIN/3.44007e-149/RecName: Full = Beta-1,3-glucan-binding protein; Short = BGBP; AltName: Full = Beta-1,3-glucan recognition protein; Short = BetaGRP; Flags: Precursor
	GAJS01022411	916	60.8	781.8	642.6	1533.0	3.4	1.0	gi|224381229|gb|ACN41858.1|/5.32372e-58/immulectin-2a [*Manduca sexta*]
	GAJS01016295	475	29.1	0.001	0.9	7.5	–4.9	12.9	gi|1042214|gb|AAB34817.1|/1.35652e-20/hemolin [*Hyalophora cecropia*]
	GAJS01018114	795	35.0	212.9	2.3	53.2	–4.0	–2.0	gi|110649252|emb|CAL25135.1|/1.8851e-50/leureptin [*Manduca sexta*]
	GAJS01007039	926	100	9.2	388.3	18.7	2.0	1.0	gi|112983062|ref|NP_001037056.1|/2.95994e-56/C-type lectin 21 [*Bombyx mori*]
	GAJS01011562	905	24.0	17.1	10.2	3.8	–1.2	–2.2	gi|307198794|gb|EFN79581.1|/1.7005e-65/Scavenger receptor class B member 1 [*Harpegnathos saltator*]
	GAJS01069548	440	963.1	2211.8	3508.2	2929.6	1.9	0.4	gi|112983550|ref|NP_001036879.1|/6.20247e-46/nimrod B [*Bombyx mori*]
	GAJS01049295	305	-	91.4	-	21.7	-	–2.0	gi|300440395|gb|ADK20132.1|/4.21981e-26/eater [*Drosophila melanogaster*]
**Extracellular signal modulators**	GAJS01024214	803	436.7	2031.0	1395.0	4882.3	1.7	1.3	gi|56418397|gb|AAV91006.1|/5.13605e-96/hemolymph proteinase 8 [*Manduca sexta*]
	GAJS01000483	1218	30.8	-	87.1	-	1.5	-	gi|56418399|gb|AAV91007.1|/6.37121e-95/hemolymph proteinase 9 [*Manduca sexta*]
	GAJS01070668	1147	148.6	276.1	300.4	201.3	1.0	–0.5	gi|56418393|gb|AAV91004.1|/1.43824e-117/hemolymph proteinase 6 [*Manduca sexta*]
	GAJS01016084	413	2.1	24.1	6.5	11.1	1.6	–1.1	gi|56418411|gb|AAV91013.1|/5.07008e-43/hemolymph proteinase 16 [*Manduca sexta*]
	GAJS01070369	667	112.9	391.7	364.6	562.5	1.7	0.5	gi|56418423|gb|AAV91019.1|/2.99863e-34/hemolymph proteinase 21 [*Manduca sexta*]
	GAJS01020826	480	240.0	1292.2	1304.3	3823.6	2.4	1.6	gi|60299968|gb|AAX18636.1|/6.65291e-31/prophenoloxidase-activating proteinase-1 [*Manduca sexta*]
	GAJS01014028	715	118.9	1732.7	514.8	1174.3	2.1	–0.6	gi|60299972|gb|AAX18637.1|/6.26969e-76/prophenoloxidase-activating proteinase-3 [*Manduca sexta*]
	GAJS01002511	656	341.7	1544.1	1789.5	4411.1	2.4	1.5	gi|156968401|gb|ABU98654.1|/8.88775e-92/prophenoloxidase activating enzyme [*Helicoverpa armigera*]
	GAJS01011386	1379	6.9	330.5	24.2	537.1	1.8	0.7	gi|63207765|gb|AAV91432.2|/1.35336e-77/serine protease 1 [*Lonomia obliqua*]
	GAJS01001284	1181	25.6	0.001	57.4	0.4	1.2	8.8	gi|114053005|ref|NP_001040537.1|/2.03846e-98/serine protease 7 [*Bombyx mori*]
	GAJS01018021	1108	5.1	0.6	1.0	0.001	–2.3	–9.3	gi|112982842|ref|NP_001036891.1|/4.18254e-151/clip domain serine protease 4 [*Bombyx mori*]
	GAJS01016886	1063	8.1	77.0	1.9	18.2	–2.1	–2.1	gi|4530064|gb|AAD21841.1|/2.49294e-12/trypsin-like serine protease [*Ctenocephalides felis*]
	GAJS01016641	1450	152.8	24.0	65.5	4.0	–1.2	–2.6	gi|114053005|ref|NP_001040537.1|/6.8406e-56/serine protease 7 [*Bombyx mori*]
	GAJS01023310	1653	8.5	12.2	4.1	3.4	–1.1	–1.9	gi|91078858|ref|XP_972061.1|/2.64066e-145/PREDICTED: similar to thymus-specific serine protease [*Tribolium castaneum*]
	GAJS01023586	940	199.0	212.8	1218.9	303.9	2.6	0.5	gi|114052256|ref|NP_001040462.1|/1.17721e-108/serine proteinase-like protein [*Bombyx mori*]
	GAJS01013237	447	0.001		50.1		15.6		gi|158121989|gb|ABW17156.1|/2.51942e-18/serine protease inhibitor 1b [*Choristoneura fumiferana*]
	GAJS01070177	575	149.7	360.5	976.6	549.8	2.7	0.6	gi|114051043|ref|NP_001040318.1|/4.00557e-73/serine protease inhibitor 3 [*Bombyx mori*]
	GAJS01068573	353	377.8	3.5	1547.0	11.5	2.0	1.7	gi|226342878|ref|NP_001139701.1|/5.43629e-29/serine protease inhibitor 7 [*Bombyx mori*]
	GAJS01013046	256	275.6	0.0	1033.8	7.0	1.9	12.8	gi|226342878|ref|NP_001139701.1|/3.74696e-14/serine protease inhibitor 7 [*Bombyx mori*]
	GAJS01013360	790	634.2	2.2	2075.0	9.0	1.7	2.0	gi|226342878|ref|NP_001139701.1|/6.57697e-64/serine protease inhibitor 7 [*Bombyx mori*]
	GAJS01004151	553	95.2	93.5	22.7	33.1	–2.1	–1.5	gi|112983872|ref|NP_001036857.1|/4.68659e-36/serine protease inhibitor 12 [*Bombyx mori*]
	GAJS01022789	667	94.8	99.1	32.9	35.5	–1.5	–1.5	gi|112983872|ref|NP_001036857.1|/4.02467e-55/serine protease inhibitor 12 [*Bombyx mori*]
	GAJS01070731	1977	90.8	100.5	33.1	56.6	–1.5	–0.8	gi|226342886|ref|NP_001139705.1|/2.03235e-118/serine protease inhibitor 13 [*Bombyx mori*]
	GAJS01005386	1806	6.7	20.7	11.3	6.3	0.8	–1.7	gi|226342888|ref|NP_001139706.1|/1.88912e-107/serine protease inhibitor 14 [*Bombyx mori*]
	GAJS01006709	937	374.4	824.4	64.0	408.8	–2.5	–1.0	gi|270358644|gb|ACZ81437.1|/6.20295e-94/serpin-4 [*Bombyx mori*]
	GAJS01070645	1042	147.4	320.3	85.7	121.3	–0.8	–1.4	gi|112983210|ref|NP_001037021.1|/5.78423e-123/serine protease inhibitor 2 [*Bombyx mori*]
	GAJS01006255	670	10.0	20.4	27.4	3.0	1.5	–2.7	gi|307563506|gb|ADN52338.1|/9.99209e-62/serpin-2 [*Bombyx mandarina*]
	GAJS01015946	303	68.4	121.3	42.9	41.1	–0.7	–1.6	gi|307563506|gb|ADN52338.1|/9.77349e-07/serpin-2 [*Bombyx mandarina*]
	GAJS01017332	987	16.2	40.1	9.1	19.3	–0.8	–1.1	gi|45594232|gb|AAS68507.1|/7.53644e-93/serpin-5A [*Manduca sexta*]
	GAJS01021859	1257	-	283.6	-	103.0	-	–1.5	gi|38564807|gb|AAR23825.1|/0/dopa-decarboxylase [*Antheraea pernyi*]
	GAJS01023812	251	39.5	89.2	22.3	41.5	–0.8	–1.1	gi|15824041|dbj|BAB68549.1|/6.8687e-16/dopa decarboxylase [*Mythimna separata*]
	GAJS01021025	659	14.1	25.3	17.7	197.2	0.3	3.0	gi|74038580|dbj|BAE43824.1|/4.41671e-123/tyrosine hydroxylase [*Papilio xuthus*]
	GAJS01006668	572	14.3	32.9	27.0	245.0	0.9	2.9	gi|223890158|ref|NP_001138794.1|/7.68621e-77/tyrosine hydroxylase [*Bombyx mori*]
	GAJS01016627	809	11.2	20.2	11.9	148.1	0.1	2.9	gi|114842171|dbj|BAF32573.1|/5.94654e-100/tyrosine hydroxylase [*Mythimna separata*]
**Intracellular signaling transducers**	GAJS01003557	344	30.7	20.1	87.2	24.4	1.5	0.3	gi|307177665|gb|EFN66711.1|/8.23419e-06/Protein spaetzle [*Camponotus floridanus*]
	GAJS01014500	517	15.4	30.9	10.0	8.4	–0.6	–1.9	gi|307210111|gb|EFN86808.1|/2.29194e-12/Protein toll [*Harpegnathos saltator*]
	GAJS01021639	557	21.3	39.5	17.3	12.3	–0.3	–1.7	gi|270002878|gb|EEZ99325.1|/1.8901e-16/toll-like protein [*Tribolium castaneum*]
	GAJS01022106	1546	15.5	23.9	5.4	10.7	–1.5	–1.2	gi|270009272|gb|EFA05720.1|/6.50266e-79/pelle [*Tribolium castaneum*]
	GAJS01022818	1039	247.8	45.6	82.5	23.7	–1.6	–0.9	gi|289629214|ref|NP_001166191.1|/4.19369e-65/cactus [*Bombyx mori*]
**Effectors**	GAJS01018460	443	26.3	2775.7	76.8	1566.3	1.5	–0.8	gi|14517795|gb|AAK64363.1|AF336289_1/3.84673e-67/prophenoloxidase [*Galleria mellonella*]
	GAJS01004863	470	33.1	7.2	94.3	11.2	1.5	0.6	gi|34556399|gb|AAQ75026.1|/1.00233e-81/prophenoloxidase subunit 2 [*Galleria mellonella*]
	GAJS01019975	394	36.7	2440.7	79.6	2686.1	1.1	0.1	gi|113376731|gb|ABC59699.2|/9.77758e-47/prophenoloxidase [*Ostrinia furnacalis*]
	GAJS01004480	525	30.4	3779.7	104.9	3534.7	1.8	–0.1	gi|34556399|gb|AAQ75026.1|/1.13882e-22/prophenoloxidase subunit 2 [*Galleria mellonella*]
	GAJS01048130	288	151.3	95.6	138.4	626.1	–0.1	2.7	gi|239579429|gb|ACR82291.1|/5.42012e-21/attacin-like antimicrobial protein [*Papilio xuthus*]
	GAJS01064740	241	159.4	35.4	9617.7	81.3	5.9	1.2	gi|283100188|gb|ADB08384.1|/1.35226e-11/attacin [*Bombyx mori*]
	GAJS01063163	219	657.2	2953.0	1134.9	1994.1	0.8	–0.6	gi|239579431|gb|ACR82292.1|/2.7697e-09/cecropin [*Papilio xuthus*]
	GAJS01018928	667	27.5	1590.8	97.0	2941.6	1.8	0.9	gi|260765457|gb|ACX49766.1|/9.0706e-15/defensin-like protein 1 [*Manduca sexta*]
	GAJS01052838	386	1.7	14.3	0.6	54.7	–1.5	1.9	gi|146737994|gb|ABQ42575.1|/1.58609e-12/moricin-like peptide C1 [*Galleria mellonella*]
	GAJS01007618	449	85.5	9.1	434.3	7.9	2.3	–0.2	gi|145286562|gb|ABP52098.1|/2.67606e-28/lysozyme-like protein 1 [*Antheraea mylitta*]
	GAJS01020045	361	0.6	26.6	6.2	8.5	3.4	–1.7	gi|1705743|sp|P50722.1|CE3F_HYPCU/7.29346e-18/RecName: Full = Hyphancin-3F; AltName: Full = Cecropin-A2; AltName: Full = Hyphancin-IIIF; Flags: Precursor
	GAJS01058138	328	55.9	296.8	2589.9	162.8	5.5	–0.9	gi|171262307|gb|ACB45565.1|/7.93671e-08/gloverin-like protein [*Antheraea pernyi*]

**Table 4:** A list of *Chilo suppressalis* development- and non-immune metabolism-related transcript that were differentially expressed after parasitization by *Cotesia chilonis*.

Gene ID	Nt. Length	RPKM				log2 Ratio		Blast results
		Cs-FB	Cs-HC	PCs-FB	PCs-HC	PCs-FB/Cs-FB	PCs-HC/Cs-HC	
GAJS01017916	791	27.6	0.001	85.5	0.6	1.6	9.3	gi|7327277|gb|AAB25736.2|/2.60437e-36/juvenile hormone binding protein [*Manduca sexta*]
GAJS01005072	420	10.8	3.0	86.9	4.8	3.0	0.7	gi|112983178|ref|NP_001037027.1|/3.48684e-30/juvenile hormone esterase 1 [*Bombyx mori*]
GAJS01008229	916	54.9	1.9	351.5	18.3	2.7	3.2	gi|157908523|dbj|BAF81491.1|/4.2949e-108/juvenile hormone epoxide hydrolase [*Bombyx mori*]
GAJS01070607	945	178.8	20.5	833.3	41.7	2.2	1.0	gi|90025232|gb|ABD85119.1|/4.47688e-124/juvenile hormone epoxide hydrolase [*Spodoptera exigua*]
GAJS01010696	621	539.6	0.9	4.7	2.5	–6.8	1.5	gi|409430|gb|AAA29312.1|/3.3712e-26/ecdysteroid regulated protein [*Manduca sexta*]
GAJS01008390	822	80.8	2.2	7056.6	344.4	6.4	7.3	gi|110743533|dbj|BAE98324.1|/6.68194e-139/methionine-rich storage protein [*Chilo suppressalis*]
GAJS01064463	237	2043.0	5.2	6776.7	148.1	1.7	4.8	gi|138369030|gb|ABO27098.2|/1.49122e-23/storage protein 2 [*Omphisa fuscidentalis*]
GAJS01002551	1478	19.4	61.6	776.7	25.6	5.3	–1.3	gi|2498144|sp|Q25490.1|APLP_MANSE/1.95917e-144/RecName: Full = Apolipophorins; Contains: RecName: Full = Apolipophorin-2; AltName: Full = Apolipophorin II; AltName: Full = apoLp-2; Contains: RecName: Full = Apolipophorin-1; AltName: Full = Apolipophorin I; AltName: Full = apoLp-1; Flags: Precursor
GAJS01001502	998	0.9	0.2	22.4	0.8	4.7	2.1	gi|197209944|ref|NP_001127736.1|/3.39584e-132/neuropeptide receptor A1 [*Bombyx mori*]
GAJS01016991	1563	15.2	0.9	70.3	1.0	2.2	0.1	gi|197209908|ref|NP_001127718.1|/2.31006e-167/neuropeptide receptor A20 [*Bombyx mori*]
GAJS01018758	1068	71.9	3.0	729.2	10.7	3.3	1.8	gi|266634534|dbj|BAI49425.1|/8.03098e-152/neuroglian [*Mythimna separata*]
GAJS01070434	714	51.7	3.5	856.4	18.9	4.1	2.4	gi|1708635|gb|AAC47451.1|/1.12725e-93/neuroglian [*Manduca sexta*]
GAJS01020317	555	29.9	3.8	296.1	73.3	3.3	4.3	gi|301070148|gb|ADK55520.1|/2.177e-33/small heat shock protein [*Spodoptera litura*]
GAJS01023701	875	139.3	8.1	425.8	665.9	1.6	6.4	gi|297718725|gb|ADI50267.1|/1.07322e-137/heat shock protein 70 [*Antheraea pernyi*]
GAJS01024045	311	80.5	24.0	241.9	168.5	1.6	2.8	gi|99653648|dbj|BAE94664.1|/6.07e-17/small heat shock protein 19.7 [*Chilo suppressalis*]
GAJS01053750	424	17.3	11.3	1.6	0.6	–3.4	–4.2	gi|193580127|ref|XP_001945416.1|/1.68031e-06/PREDICTED: similar to juvenile hormone-inducible protein 26 [*Acyrthosiphon pisum*]
GAJS01001291	2192	18.4	0	2.2	0	–3.0	0	gi|197209940|ref|NP_001127734.1|/0/neuropeptide receptor B3 [*Bombyx mori*]
GAJS01005512	1526	285.6	39.2	26.3	11.0	–3.4	–1.8	gi|307210784|gb|EFN87167.1|/3.0189e-47/G-protein coupled receptor Mth2 [*Harpegnathos saltator*]
GAJS01016543	1607	8.5	1897.7	6.3	269.4	–0.4	–2.8	gi|83583697|gb|ABC24708.1|/2.64791e-49/G protein-coupled receptor [*Spodoptera frugiperda*]
GAJS01011182	1789	8.7	3.8	1.3	0.7	–2.8	–2.4	gi|194440587|dbj|BAG65666.1|/0/epidermal growth factor receptor [*Gryllus bimaculatus*]
GAJS01011173	1191	55.3	78.9	5.5	36.1	–3.3	–1.1	gi|114051177|ref|NP_001040390.1|/1.73332e-121/syntaxin 5A [*Bombyx mori*]
GAJS01000028	2027	759.5	1.3	314.8	2.4	–1.3	0.9	gi|84095074|dbj|BAE66652.1|/0/phenylalanine hydroxylase [*Papilio xuthus*]
GAJS01022981	553	14.8	8.0	0.8	0.5	–4.2	–4.1	gi|298204367|gb|ADI61832.1|/2.75392e-28/endonuclease-reverse transcriptase [*Bombyx mori*]
GAJS01010928	1812	412.5	42.1	122.2	8.0	–1.8	–2.4	gi|307611929|ref|NP_001182631.1|/0/sugar transporter protein 3 [*Bombyx mori*]
GAJS01023215	956	134.3	40.3	24.1	16.8	–2.5	–1.3	gi|193627460|ref|XP_001947286.1|/4.10075e-40/PREDICTED: similar to torso-like protein [*Acyrthosiphon pisum*]
GAJS01012242	770	69.8	30.7	1.7	11.2	–5.3	–1.4	gi|157127009|ref|XP_001654758.1|/4.04297e-103/heat shock protein [*Aedes aegypti*]
GAJS01056369	1127	0.4	28.8	0.4	7.7	0.1	–1.9	gi|307171282|gb|EFN63207.1|/4.29844e-42/Insulin receptor [*Camponotus floridanus*]
GAJS01055686	613	39.4	20.9	41.6	5.4	0.1	–2.0	gi|189238570|ref|XP_969918.2|/9.47611e-18/PREDICTED: similar to sugar transporter [*Tribolium castaneum*]
GAJS01018131	1125	17.8	63.8	14.7	22.6	–0.3	–1.5	gi|157136674|ref|XP_001663817.1|/3.04495e-80/sugar transporter [*Aedes aegypti*]
GAJS01001647	1034	46.7	0.2	2.2	0.2	–4.4	0.5	gi|223671143|tpd|FAA00523.1|/1.21732e-40/TPA: putative cuticle protein [*Bombyx mori*]

In insects, pattern recognition receptors (PRRs) make up the surveillance mechanism and recognize pathogen-associated molecular patterns (PAMPs), associated with microbial pathogens or cellular stress. Hemolin is a highly inducible PRR that recognizes the lipopolysaccharide (LPS) component of Gram-negative bacteria in *Manduca sexta* [37], [38]. This gene (GAJS01016295) was down-regulated (log_2_ Ratio  =  –4.9) in fatbody and up-regulated (log_2_ Ratio  =  12.9) in hemocytes (Table 3). Although PRRs are up-regulated by infections [38], these genes can be suppressed by parasitoid venom [20], [21] or PDVs [39]. In our results, certain PRRs included peptidoglycan recognition protein B (GAJS01023399, PF/CF: –1.2), hemicentin 1 (GAJS01000005, PH/CH: –4.5), leureptin (GAJS01018114, PF/CF: –4.5) and Scavenger receptor class B (GAJS01011562, PH/CH: –2.2) were down-regulated. Other genes including β-1, 3-glucan-binding protein (GAJS01005743, PF/CF: 5.7) and immulectin-2a (GAJS01022411, PF/CF: 3.4) were up-regulated. These data indicate that wasp-associated factors of *C. chilonis* influence components of the host immune system.

Extracellular signal transduction is critical for homeostatic processes, including immunity, in insects. Hemolymph proteinases (HPs) form enzyme cascades to detect pathogen-PRR complexes and activate precursors of defense proteins, such as prophenoloxidase (PPO), spätzle, serine proteinase homology (SPH) and plasmatocyte-spreading peptide (PSP) by limited proteolysis [38], [40]. In *Manduca sexta*, 22 HPs genes were reported [41], [42], and we found ten HPs in the transcriptome of *C. suppressalis* : HP5 (log_2_ Ratio PH/CH: –3.3), HP6 (log_2_ Ratio PF/CF: 1.0), HP8 (log_2_ Ratio PF/CF: 1.7; log_2_ Ratio PH/CH: 1.3), HP9 (log_2_ Ratio PF/CF: 3.1), HP16 (log_2_ Ratio PF/CF: 1.6; log_2_ Ratio PH/CH: –1.1), HP17, HP19, HP21(log_2_ Ratio PF/CF: 1.7), PAP1 (log_2_ Ratio PF/CF: 2.4; log_2_ Ratio PH/CH: 1.6) and PAP3 (log_2_ Ratio PF/CF: 2.1) (Table 3). Most of them were up-regulated by parasitization except for HP5, which was down-regulated in parasitized hemocytes. Among of them, we obtained complete open reading frames (ORFs) for HP5, HP6 and HP8.

In insects, PPO is activated upon invasion or injury, which results in localized melanization of the wound region and/or melanotic capsules capturing invading microorganisms and parasites [12], [43]. After parasitization, transcripts encoding two PPOs were up-regulated in fatbody and hemocytes (Table 3). Consistent with this study, cDNA microarray analysis of *Spodoptera frugiperda* fatbody and hemocytes 24 hours after *Hyposoter didymator* Ichnovirus (HdIV) and *Microplitis demolitor* Bracovirus (MdBV) injection revealed the up-regulation of PPO-1 and -2 [36]. In *M. sexta*, PPO activation requires three PPO-activating proteinase (PAP) and two SPHs simultaneously [44]–[46]. We identified two PAP (PAP1 and PAP3) genes and two SPH genes: one is SPH2 (GAJS01023586, log_2_ Ratio PF/CF: 2.6). The other is a full length of masquerade-like serine proteinase (GAJS01011460) which did not changed significantly and its ortholog in *P. rapae* is up-regulated after parasitization by *P. puparum* [47]. Functions of serine proteinases are modulated by SPHs and by serine protease inhibitors (serpins). Some members of the serpin superfamily regulate serine proteinase activities through forming covalent complexes with their cognate enzymes [48]. A proteomics analysis showed that mRNA encoding serpin2 and its protein were suppressed in *P. xylostella* larvae following parasitization by *Cotesia plutellae* [49]. Beck *et al*. [50] reported that the ovarian calyx fluid of the ichneumonid endoparasitoid *Venturia canescens* has a putative serpin activity to suppress the host immune system. In our work with the rice borer, we identified three up-regulated and three down-regulated serpins in the fatbody and one up-regulated and five down-regulated serpins in the hemocytes (Table 3). In the fatbody, Serpin1b (GAJS01013237, log_2_ Ratio PF/CF: 15.6) [51], serpin3 (GAJS01070177, log_2_ Ratio PF/CF: 2.7) and serpin7 (three Unigenes, log_2_ Ratio PF/CF: 2.0, 1.9, 1.7) were up-regulated. Serpin4 (GAJS01006709, log_2_ Ratio PF/CF: –2.5), serpin12 (two Unigenes, log_2_ Ratio PF/CF: –2.1, –1.5) and serpin13 (GAJS01070731, log_2_ Ratio PF/CF: –1.5) were down-regulated. In the hemocytes, only serpin7 (GAJS01013360, log_2_ Ratio PH/CH: 2.0) was up-regulated. Serpin2 (three unigenes, log_2_ Ratio PH/CH: –1.4, –2.7, –1.6), serpin5A (GAJS01017332, log_2_ Ratio PH/CH: –1.1), serpin4 (GAJS01006709, log_2_ Ratio PH/CH: –1.1), serpin12 (two Unigenes, log_2_ Ratio PF/CF: –1.7, –1.5) and serpin14 (GAJS01005386, log_2_ Ratio PF/CF: –1.7) were down-regulated.

There are two pathways for pathogen recognition and signal transduction, a PRR-SP system in insect plasma (e.g., spätzle processing for Toll activation) or binding to PRRs on the surface of immune tissues/cells (e.g., PGRP-LC binding for Imd activation in *Drosophila*). As shown in Table 3, transcripts of most Toll and Imd pathway proteins, such as Relish, Pelle, Cactus and Toll receptor, were influenced by parasitization. These include Toll proteins (GAJS01021639, log_2_ Ratio PF/CF: –1.7) and Pelle (GAJS01022106, log_2_ Ratio PF/CF: –1.5, log_2_ Ratio PH/CH: –1.2) were down-regulated by parasitization (Table 3), which differs from *P. xylostella* after parasitization by *D. semiclausum* [12]. Overproduction of effector proteins, particularly anti-microbial peptides (AMPs), that immobilize pathogens, block their proliferation, or directly kill them is a hallmark of insect immunity [38], [52]. Consistent with this notion, we have detected some AMPs and lysozyme. Most of them were up-regulated by parasitization (Table 3). However, it’s worth pointing out that some immune response, like AMP genes, may be induced only by a puncture. Hence, some of the presented results may not be related to parasitism.

We also recorded changes in other proteins that influence immune responses in other moths such as tyrosine hydroxylase and dopa decarboxylase (Table 3). The general finding is that expression of tyrosine hydroxylase and dopa decarboxylase is significantly induced following infection [12], [53], while we found that tyrosine hydroxylase was up-regulated and dopa decarboxylase was down-regulated (Table 3).

### Transcripts related to development and metabolism

Our data indicates that parasitism leads to up-regulation of genes associated with JH binding or degradation. JHBP (GAJS01017916, log_2_ Ratio PF/CF: 1.6), JHE (GAJS01005072, log_2_ Ratio PF/CF: 1.6) and JHEH (GAJS01070607, log_2_ Ratio PF/CF: 1.6) (Table 4). JHEH transcript levels were down-regulated more than 2-fold in *P. xylostella* after parasitization by *D. semiclausum* [12]. Generally, JH is maintained at high levels during parasitoid larval development [5], [54]–[56]. Our findings run otherwise, with increases in JHE and JHEH transcript levels. This may be another example of the wide variation in molecular details of insect host-parasitoid relationships.

Parasitization of *P. xylostella* by *D. semiclausum* leads to down-regulation of genes associated with ecdysteroid activities [12]. Our data support this view as the transcript level of ecdysteroid regulated protein (GAJS01010696, log2 Ratio PF/CF: –6.8) was down-regulated in parasitized larvae (Table 4). In general, parasitization leads to reductions in ecdysteroid titres and decreases in ecdysteroid regulated proteins [43], [54], [55], [57]. We found that expression of methionine-rich storage protein (MRSP), a diapause-associated protein [58], was up-regulated (GAJS01008390, log_2_ Ratio PF/CF: 6.4; log_2_ Ratio PH/CH: 7.3) in parasitized larvae (Table 4). It indicates the crosstalk between regulatory pathways in insect diapause and in parasitoid-regulated host development. We also found that the expression of transcripts encoding a number of G-protein coupled receptors (GPCRs) was affected by parasitization. For example, allatostatin receptor (GAJS01001502, log_2_ Ratio PF/CF: 4.7; log_2_ Ratio PH/CH: 2.1), neuropeptide A20 (GAJS01016991, log_2_ Ratio PF/CF: 2.2), neuropeptide B3 (GAJS01001291, log_2_ Ratio PF/CF: –3.0) and Methuselah (Mth) (GAJS01005512, log_2_ Ratio PF/CF: –3.4; log_2_ Ratio PH/CH: –1.8) were up- or down-regulated by parasitization (Table 4). We found that transcripts for the allatostatin receptor were highly expressed and it was reported that activation of allatostatin A-expressing neuron promotes food aversion and/or exerts an inhibitory influence on the motivation to feed in adult *Drosophila* [59]. Reduced food consumption was also found in parasitized larvae in our system [17]. There might be relationships among some GPCRs. Mth is a class B secretin-like GPCR and down-regulation of *Mth* increases the life span of *D. melanogaster* [60]–[63]. We infer that decreased Mth transcription levels may help elongate the lifespan of parasitized larvae, as seen elsewhere [1].

### Validation of candidate genes

**Figure 5:**
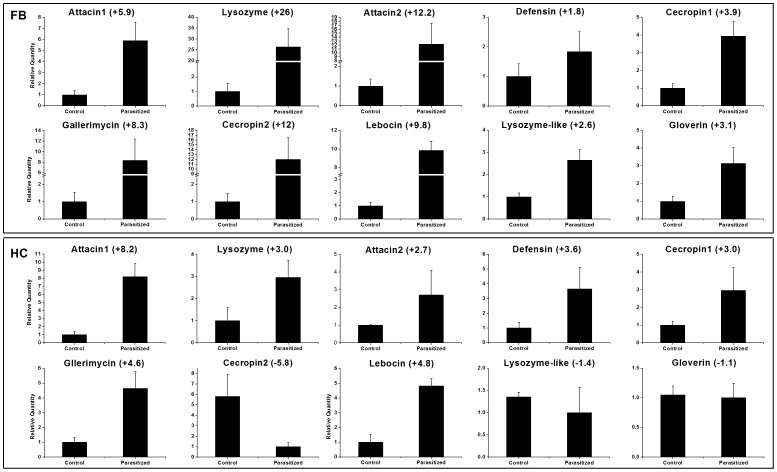
Anti-microbial peptides transcript levels in fatbody and hemocytes of non-parasitized (control) and parasitized *Chilo suppressalis* larvae. The histograms show the means ± SEM, *n*  =  3 biologically independent experiments. Fold changes are shown in brackets.

**Figure 6:**
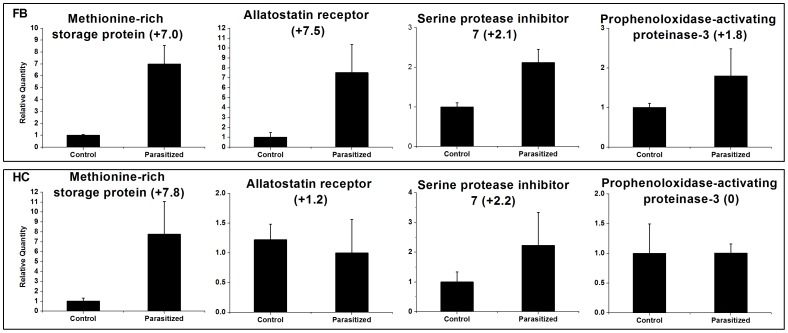
qRT-PCR analysis of four selected genes from *Chilo suppressalis* transcriptome. Error bars indicate standard deviations of averages from three replicates. Fold changes are shown in brackets.

**Figure 7:**
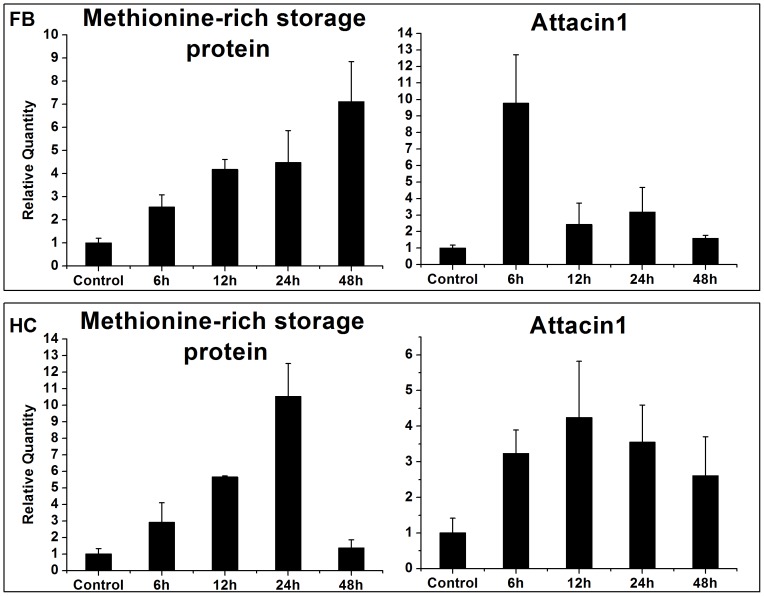
qRT-PCR analysis of expression levels of two selected genes in *Chilo suppressalis* larvae at four time points after parasitization with *Cotesia chilonis*. Error bars indicate standard deviations of averages from three replicates. Fold changes are shown in brackets..

To validate our RNA-seq data, we performed qRT-PCR on 14 selected immune- and development-related genes, including ten anti-microbial peptides, MRSP, neuropeptide A1, serine protease inhibitor 7 and PAP 3 (Figure 5, 6). The sequences of the primers used are given in Table 5. Our results are consistent with the RNA-seq profiles showing similar trends in up- or down-regulation (or steady-state) of host genes with little difference (Figure 5, 6). For example, based on RNA-seq analysis, attacin1 (↑ 6-fold), defensin (↑ 2-fold), MRSP (↑ 6-fold) and PAP 3 (↑ 2-fold) were up-regulated in fatbody (Table 3, 4) and showed parallel changes in our qRT-PCR analysis (Figure 5, 6). These data are based on pools of RNA from various time points. To investigate the expression of host genes at different periods after parasitization, we isolated RNA from 4^th^ instar larvae at selected time points after parasitization, and analyzed two associated genes, MRSP and attacin 1 (Figure 7). The expression of MRSP increased with time through 48 h following parasitization in fatbody; whereas transcripts of MRSP increased to a maximum at 24 h and then returned to the basal level by 48 h in hemocytes. Fatbody expression of attacin 1 peaked at 6 h p.p. and at 12 h in hemocytes.

**Table 5:** The primers used in this study.

Gene	Forward primer	Reverse primer
Allatostatin receptor	GCTTCGCTACTCCAAAATGC	GGTGGCAGACCGCTATGTAT
Methionine-rich storage protein	TCCATTCAAGGTCACGATCA	CTTGCCGGTGTCCAGTTTAT
Serine protease inhibitor 7	AAGCGGAGTTGAGGTTCAGA	GGCAGTCACTTGTTTGACGA
Prophenoloxidase-activating proteinase-3	AATTAGGCACACCGAGCAAC	TCAGGGCTGTACTGCTGATG
Attacin1	GCACAGCCAGAATCATAACG	GATACTGAGAGCCCGTGACC
Attacin2	CTGGTGGTATAACGGCGACT	CGCTGACCTGATCCCTGTAT
Cecropin1	TCTTCAAGAAAATCGAGAAG	TGAGTATTCTCTTTGGCATT
Cecropin2	TTGTTTTTCGTGTTCGCTTG	AAATTCAACGTCCCTTCACG
Defensin	GCGCGTAATACCGTTTGTCT	CGCAAAGGCCATAGGAATAG
Gloverin	GATGTCAGCAAGCAGATAGGC	CGAAAGCACCCAGAAAAAGA
Lysozyme-like	TGCGCTCAGCTGATCTTCTA	CCTTCTCGCCAATCTACGTC
Lysozyme	GGGACCCGTTACTGTTGGT	CTGGCAATGCGAAGCTAAA
Gallerimycin	AATACCCGGTGCACACAAAC	ATACAGGCGCATCCGTTAAG
Lebocin	ATGTTGCGAAGAGCGAGTTT	GCCCGATTTACATCATCACC
18S rRNA	TCGAGCCGCACGAGATTGAGCA	CAAAGGGCAGGGACGTAATCAAC

### Cotesia chilonis BV transcripts

PDVs are categorized into two genera: BV and IVs, which are associated with braconid and ichneumonid wasps, respectively. To date, the genomes of five BVs [*Cotesia congregata* BV (CcBV), *Cotesia vestalis* (CvBV), MdBV, *Glyptapanteles indiensis* BV (GiBV) and *Glyptapanteles flavicoxis* BV (GfBV)] and three IVs [*Campoletis sonorensis* IV (CsIV), *Glypta fumiferanae* (GfIV) and *Hyposoter fugitivus* IV(HfIV)] have been fully sequenced [15], [64]–[66], and many genes of likely wasp origin have been determined which encode proteins involved in changing host physiology [67]. Some conserved gene families, including ankyrin, BEN domain-coding protein, CrV1-like protein, cystatin, early-expressed protein (EP), lectin and protein tyrosine phosphatases (PTPs), have been reported in most BV genomes [15].

We detected a range of *CchBV* genes in parasitized larvae. Based on our analysis, 19 unique sequences were identified from six PDV gene families including ankyrin, CrV1 protein, cystatin, EP, lectin and PTPs (Table 6). Besides these, eight other hypothetical proteins with unknown function were also found in parasitized larvae, and showed more than 47% similarity with some parts of BV reference genomes. The presence of multiple sequences for each PDV gene family is expected. We identified 2 segments with ankyrin domain, which are commonly shared by BV and IV, and 2 CrV1 transcripts, which showed high similarity (> 90%) with *Cotesia sesamiae* CrV1 protein [68] (Table 6). These two transcripts may belong to one gene. We detected two EP-like proteins. The EP genes encode secreted, glycosylated proteins that are expressed within 30 min in host and accumulate to comprise over 10% of total hemolymph proteins by 24 h p.p. [69], which could induce significant reduction in total hemocyte numbers and suppress host immune response presumably by its hemolytic activity during parasitization [70]. Three PTPs transcripts, which have 33 members as the largest family in CvBV [15], were also found in the parasitized larvae. Previous studies suggested that some but not all PTPs function as a phagocytic inhibitor or apoptosis inducer, which plays an important role in suppressing insect immune cell [71], [72]. We found one lectin and one cystatin transcript in *CchBV*. All these proteins were also found in other BVs, like CvBV, CcBV and CrBV.

**Table 6:** *Cotesia chilonis* bracovirus (BV) transcripts which were detected in parasitized *Chilo suppressalis* larvae.

Protein	Gene ID	Nt. Length	Blast results	Max score	Total score	Query coverage	E-value	Max identity	Conserved Domains
Ankyrin	GAJS01020778	275	gi|332139218|gb|AEE09523.1| viral ankyrin [*Cotesia vestalis* bracovirus]	175	175	94%	1e-44	68%	Yes
Ankyrin	GAJS01052562	377	gi|190702436|gb|ACE75325.1| viral ankyrin [*Glyptapanteles indiensis*]	139	139	53%	1e-31	67%	Yes
CrV1 protein	GAJS01035159	529	gi|124558219|gb|ABN13950.1| CrV1 protein [*Cotesia sesamiae*]	496	496	98%	1e-149	90%	Yes
CrV1 protein	GAJS01035178	474	gi|124558243|gb|ABN13951.1| CrV1 protein [*Cotesia sesamiae*]	489	729	86%	1e-153	97%	Yes
Cystatin 2	GAJS01041934	233	gi|332139261|gb|AEE09558.1| cystatin 2 [*Cotesia vestalis* bracovirus]	109	109	51%	3e-23	83%	Yes
EP1-like protein	GAJS01039165	217	gi|332139198|gb|AEE09505.1| EP1-like protein [*Cotesia vestalis* bracovirus]	153	1e-37	92%	1e-37	82%	Yes
EP2-like protein	GAJS01036467	204	gi|332139170|gb|AEE09482.1| EP2-like protein [*Cotesia vestalis* bracovirus]	115	225	92%	3e-25	82%	Yes
Lectin	GAJS01070737	235	gi|332139303|gb|AEE09593.1| lectin [*Cotesia vestalis* bracovirus]	117	117	62%	1e-25	72%	Yes
Protein tyrosine phosphatase 1	GAJS01023751	346	gi|313199469|emb|CAS06603.1| protein tyrosine phosphatase [*Cotesia vestalis* bracovirus]	238	238	98%	5e-64	68%	Yes
Protein tyrosine phosphatase 2	GAJS01050386	325	gi|190343053|gb|ACE75485.1| protein tyrosine phosphatase [*Glyptapanteles indiensis* bracovirus]	150	150	72%	2e-35	67%	Yes
Protein tyrosine phosphatase 3	GAJS01052581	378	gi|190343052|gb|ACE75484.1| protein tyrosine phosphatase [*Glyptapanteles indiensis* bracovirus]	169	169	61%	3e-41	73%	Yes
Unknown Protein 1	GAJS01003889	734	gi|332139217|gb|AEE09522.1| conserved hypothetical protein [*Cotesia vestalis* bracovirus]	148	232	40%	1e-33	55%	No
Unknown Protein 2	GAJS01034489	345	gi|57659618|ref|YP_184880.1| hypothetical protein CcBV_30.6 [*Cotesia congregata* bracovirus]	186	288	93%	1e-46	65%	No
Unknown Protein 3	GAJS01041812	232	gi|117935429|gb|ABK57054.1| hypothetical protein GIP_L1_00690 [*Glyptapanteles indiensis*]	53.3	53.3	64%	3e-05	43%	No
Unknown Protein 4	GAJS01047995	287	gi|57659618|ref|YP_184880.1| hypothetical protein CcBV_30.6 [*Cotesia congregata* bracovirus]	151	492	97%	5e-36	77%	No
Unknown Protein 5	GAJS01007713	362	gi|118139723|gb|EF067323.1| *Cotesia plutellae* polydnavirus segment S22, complete sequence	120	208	43%	9e-24	79%	No
Unknown Protein 6	GAJS01036546	205	gi|394804260|gb|AFN42304.1| hypothetical protein CsmBV_7.5 [*Cotesia sesamiae* Mombasa bracovirus]	87.2	87.2	35%	3e-16	100%	No
Unknown Protein 7	GAJS01040343	224	gi|118139737|gb|ABK63323.1| hypothetical protein [*Cotesia plutellae* polydnavirus]	197	197	99%	3e-53	81%	No
Unknown Protein 8	GAJS01037505	209	gi|332139193|gb|HQ009535.1| *Cotesia vestalis* bracovirus segment c12, complete sequence	168	168	78%	1e-38	83%	No

**Figure 8:**
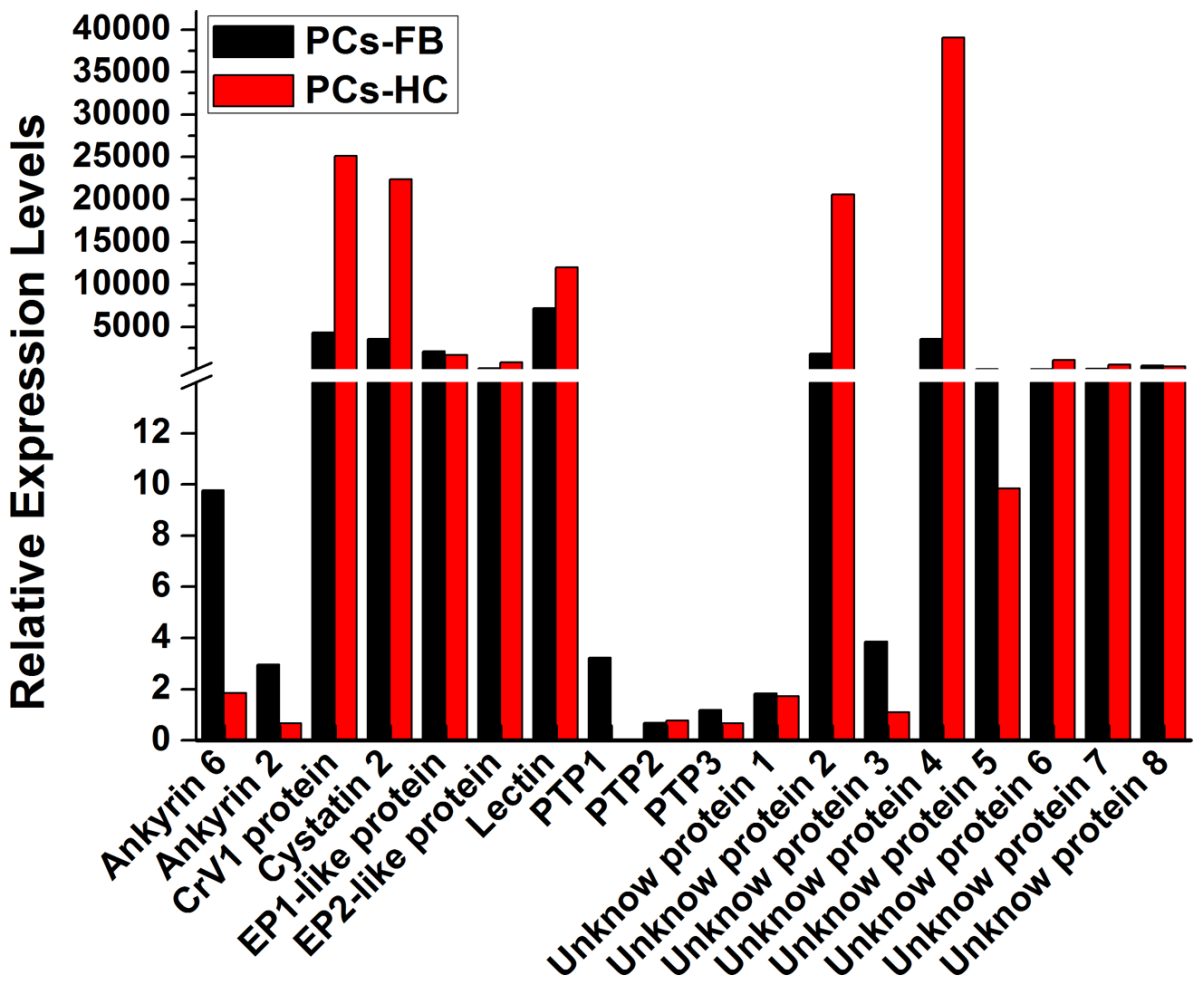
Relative gene expression values based on average read depth for all detected *Cotesia chilonis* bracovirus genes. RPKM normalized values were used to generate the data.

Transcription levels differed among different members and tissues with each gene family. Ankyrin 6, 2 and PTP1, 2, 3 had the lowest transcription levels relative to other *CchBV* genes in PCs-FB and PCs-HC (Figure 8). Ankyrin and PTP1 were mostly detected in PCs-FB. However, CrV1, cystatin 2 protein and lectin were mostly transcribed in PCs-HC and showed a higher transcription levels. EP1-like and EP2-like gene showed a parallel expression in both of infected tissues. Among the Unknown proteins, the unknown protein 2 and 4 had the highest transcription levels and expressed much more in PCs-HC (Figure 8). The reasons why these genes expressed differential in different infected tissues need more investigation.

## Conclusions

In summary, we investigated the global gene transcription profiles of fatbody and hemocytes of *C. suppressalis* in response to parasitization by *C. chilonis*. The results showed that the abundances of relatively low proportion of *C. suppressalis* transcripts were differentially expressed after parasitization by *C. chilonis*. And most of these affected genes have predicted roles in immunity, development, or detoxification. At the tissue level, our results indicated that the expressions of fat body genes were changed much more than those of hemocytes. Since we pooled the samples of four time point post-infection together, it is possible that we would loss the opportunity to assess patterns in transcriptome activity as function of sample time. Our results provide evidence for expression of 18 CchBV transcripts expressed in the host. The expression levels of these PDV genes were different at two tissues. It is also possible that CchBV gene products affect the growth and immune state of host through interactions at the protein level, such as viral proteins interacting with specific host proteins and epigenetic regulation. In addition, these viruses may also produce small non-coding RNAs that modulate host gene transcription or microRNA of host differentially expressed in response to parasitization [73]. The transcriptome data obtained in this study provides a basis for future research in this under-explored host-parasitoid interaction. Future functional studies on the identified immune-, development- and detoxification-related genes could lay the foundation for identifying hot-spots for host-parasitoid interaction, which could contribute to develop new strategies to optimize use of parasitoids for *C. suppressalis* control.

## Supporting Information

Figure S1 **Histogram presentation of Gene Ontology classification.** The results are summarized in three main categories: biological process, cellular component and molecular function. The right y-axis indicates the number of genes in a category. The left y-axis indicates the percentage of a specific category of genes in that main category. The main and specific categories are indicated on the x-axis.
(TIF)

Figure S2 **Histogram presentation of clusters of orthologous groups (COG) classification.** All putative proteins were aligned to the COG database and can be classified functionally into at least 25 molecular families.
(TIF)
