# Supplementary figures and images for: Parasitization by Cotesia chilonis Influences Gene Expression in Fatbody and Hemocytes of Chilo suppressalis
Source: PLoS One. 2013 Sep 23;8(9):e74309. doi: 10.1371/journal.pone.0074309 (PMC3781088; doi:10.1371/journal.pone.0074309)

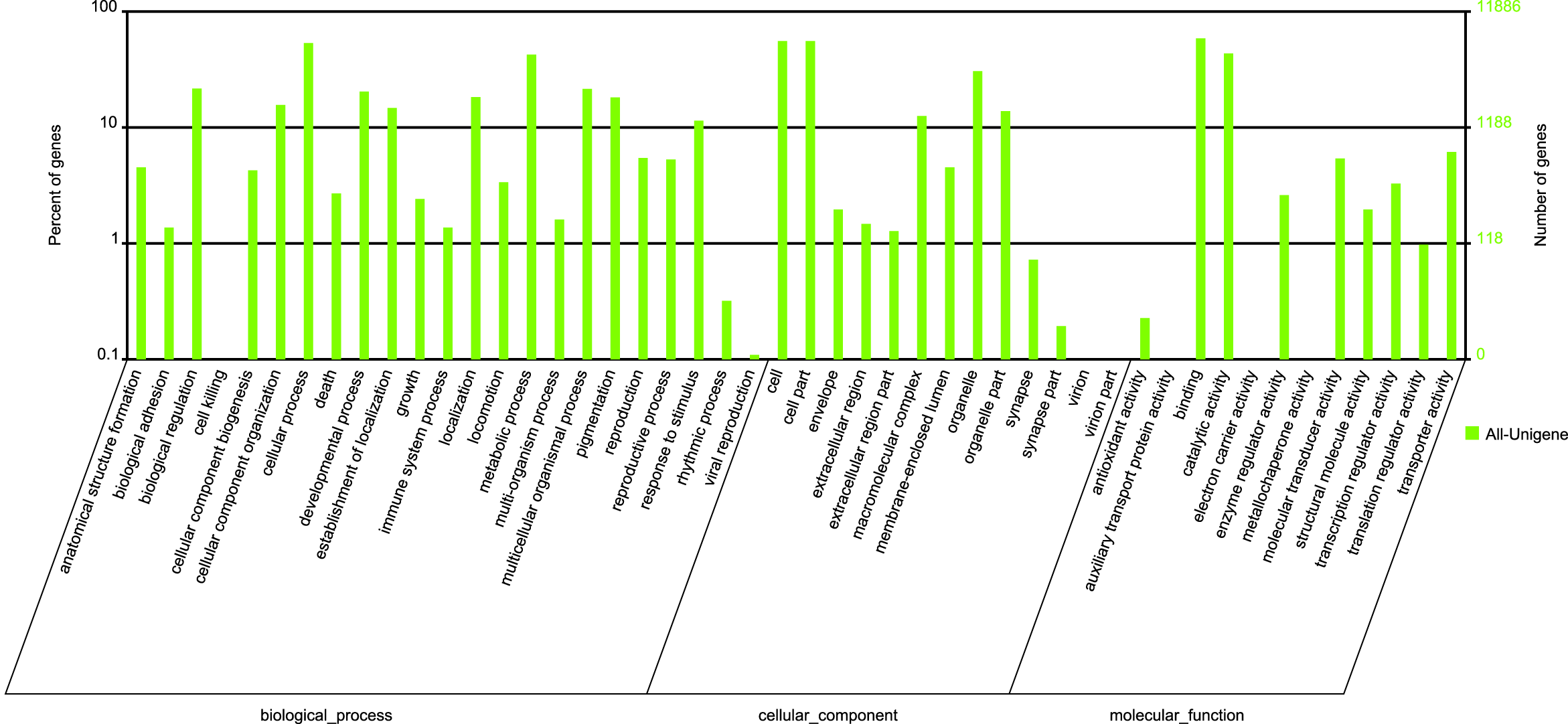

Supplement: Figure S1 — Histogram presentation of Gene Ontology classification. The results are summarized in three main categories: biological process, cellular component and molecular function. The right y-axis indicates the number of genes in a category. The left y-axis indicates the percentage of a specific category of genes in that main category. The main and specific categories are indicated on the x-axis. (TIF) [file pone.0074309.s001.tif]

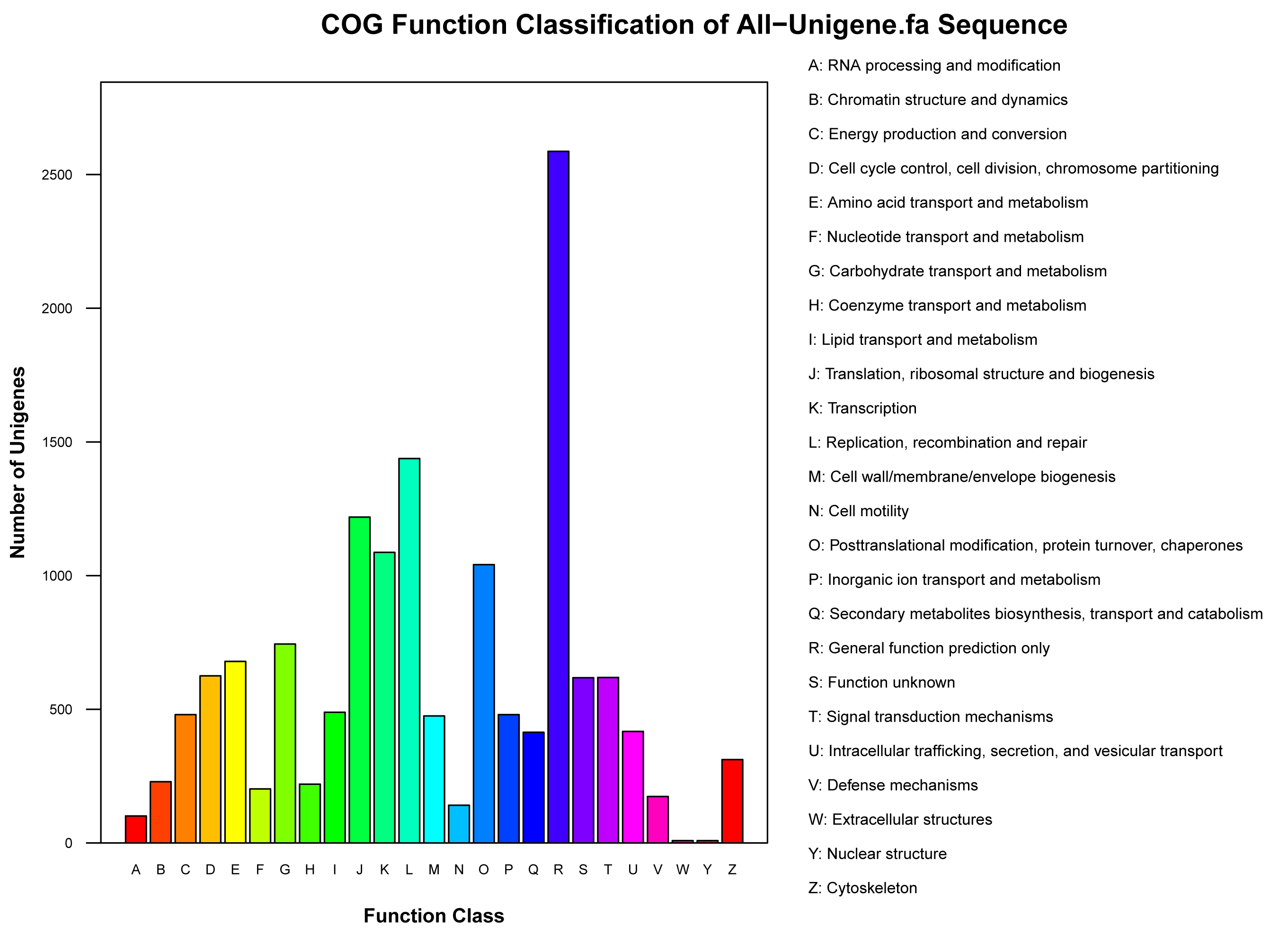

Supplement: Figure S2 — Histogram presentation of clusters of orthologous groups (COG) classification. All putative proteins were aligned to the COG database and can be classified functionally into at least 25 molecular families. (TIF) [file pone.0074309.s002.tif]
